# In-situ preparation of norepinephrine-functionalized silver nanoparticles and application for colorimetric detection of tacrolimus in plasma samples

**DOI:** 10.1016/j.heliyon.2023.e18404

**Published:** 2023-07-20

**Authors:** Zahra Golsanamlu, Jafar Soleymani, Afshin Gharekhani, Abolghasem Jouyban

**Affiliations:** a Pharmaceutical Analysis Research Center and Faculty of Pharmacy, Tabriz University of Medical Sciences, Tabriz, Iran; b Department of Pharmaceutical Chemistry, Faculty of Pharmacy, Tabriz University of Medical Sciences, Tabriz, Iran; c Department of Clinical Pharmacy (Pharmacotherapy), Faculty of Pharmacy, Sina Hospital, Tabriz University of Medical Sciences, Tabriz, Iran; d Pharmaceutical Sciences Research Center, Shahid Beheshti University of Medical Sciences, Tehran, Iran

**Keywords:** Biomedical analysis, Tacrolimus, Colorimetric, Patient real samples, Silver nanoparticles

## Abstract

Tacrolimus (Tac) is a well-documented immunosuppressive agent for the prevention of graft-vs-host diseases in several types of organ transplants. The narrow therapeutic window and the individual-variable pharmacokinetics of Tac demonstrate the importance of regular therapeutic drug monitoring (TDM) as an imperative concept for its oral medication regimens. A simple, one-step, selective, and sensitive colorimetric platform is fabricated for the determination of Tac by surface modification of the silver nanoparticles (AgNPs) via norepinephrine (NE) molecules. The attachment of NE and Tac induces the aggregation of the AgNPs, which is observed by color distinction (yellow to brown) and a noteworthy shifting of the absorption peak in the visible region. The fabricated nanoprobe can detect Tac concentrations in plasma samples in two linear ranges from 2 ng/mL to 70 ng/mL and 70 ng/mL to 1000 ng/mL with R^2^ > 0.99. The limit of detection (LOD) was calculated as low as 0.1 ng/mL. The developed method was applied for the determination of Tac in patient’s plasma samples under Tac medication therapy.

## Graphical abstract

**Figure:**
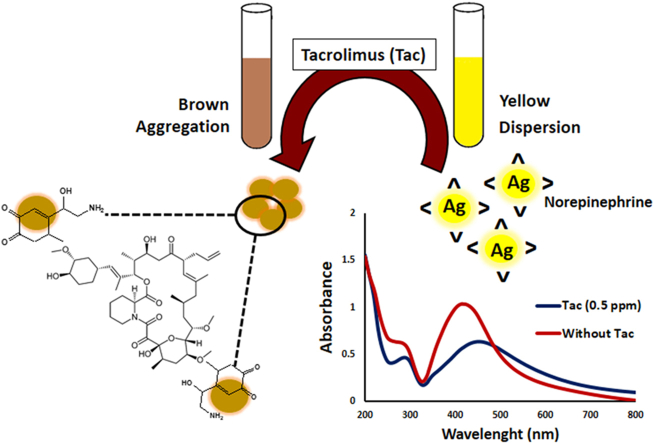

## 1 Introduction

Tacrolimus (Tac), also known as FK-506, is a well-documented immunosuppressive agent for the prevention of graft-*vs-*host disease in the follow-up medication of several types of organ transplants. The immunosuppression mechanism of Tac relates to the formation of a pentameric complex with Ca^2+^, calcineurin, and calmoduline, which inhibits the nuclear factor of activated T-cells (NFAT). NFAT is a necessary factor for the production of interleukin 2 [[1], [2], [3]]. The over-dosage administration of Tac leads to some severe consequences and toxicities, like nephrotoxicity. While the under-dosage decreases its efficacy in the prevention of transplant rejection [[4], [5], [6], [7]]. To overcome this, the whole blood level of Tac must be retained in the range of 5–20 ng/mL [8]. Additionally, the narrow therapeutic window and the individual-variable pharmacokinetics of Tac demonstrate the importance of regular therapeutic drug monitoring (TDM) as an imperative concept for its oral medication regimens [9].

So far, numerous analytical methods have been used for the quantification of Tac, such as liquid chromatography tandem mass spectrometry (LC-MS/MS) [[10], [11], [12], [13]], ultra-high performance liquid chromatography tandem mass spectrometry (UPLC-MS/MS) [[14], [15], [16]], UHPLC [17], enzyme-linked immunosorbent assay (ELISA) [18], enzyme multiplied immunoassay technique (EMIT) [19], automated microparticle enzyme immunoassay (MEIA) [20], electro-chemiluminescence immunoassay (ECLIA) [21], fluorescent aptasensor [22], and electrochemical immunosensor [9]. Some of these methods require extensive training, a high degree of technical ability, a time-consuming process, and high-cost sophisticated equipment, which are the main drawbacks for rapid and real-time routine quantifications [23]. Modern analysis technology appreciates fast, simple training, and sensitive techniques for TDM, which can be operated for on-site applications [9,[24], [25], [26], [27]]. The colorimetric sensors have advantages compared with other quick-detection methods such as electrochemical, fluorescent, etc. due to their naked-eye determination, which is more economical without requiring complicated instruments [28]. As an additional feature for simple colorimetric analysis is the possibility of their precise quantifications using smart-phone applications [29,30]. Various types of materials were utilized for colorimetric detection in different media [23,28,31]. For instance, metal nanoparticles (NPs) such as gold (Au), silver (Ag), copper (Cu), and platinum (Pt) recently appealed to global interests as favorable materials for the invention of an appropriate colorimetric nanosensor due to their distinct color through aggregation state in the presence of the target analytes [[31], [32], [33], [34], [35], [36]]. Surface Plasmon Resonance (SPR) refers to the phenomenon that the incident light reflects at an angle on the surface of the metal NPs, which causes a decrease in light intensity. As a result, a noteworthy shift in the absorption peak is observed in the visible region (390–750 nm). Today, plasmonic nanosensors offer the high-priority principle for several recent straight-mode color-based detection applications [37,38].

AgNPs are well-known nanoparticles to be inexpensive colorimetric sensors (in comparison to AuNPs) owning to their ease of color visualization, size-shape-dependent features, strong spectral shifts, high SPR properties, biocompatibility, and long-term stability [39,40]. An AgNPs colorimetric assay typically involves the color change from “bright-yellow to red”, where the red color expresses the aggregation state [39]. In addition, the surface modification of the AgNPs can effectively enhance the detection performance.

To provide a simple, selective, and sensitive analytical platform and alter the colorimetric sensing ability of AgNPs, norepinephrine (NE) has been utilized for modification of AgNPs to quantify Tac concentrations in plasma samples. NE with two hydroxyl groups as the multifunctional group modifier increases the bonding ability of the AgNPs to the target analyte. Here, the NE is not only used as a reducing agent but also helps stabilize the AgNPs. Both the functionalization of AgNPs with NE and Tac detection occur in a one-step process. The attachment of NE and Tac induced the aggregation of the AgNPs, resulting in a change in the size and absorption peak of the NPs. The developed method was applied for the determination of Tac in patients’ plasma samples with Tac oral consumption regimens.

## 2 Experimental section

### 2.1 Reagents

NE powders was provided from Sigma-Aldrich Co. (Taufkirchen, Germany). Silver nitrate (AgNO_3_) powder, sodium hydroxide (NaOH), phosphate buffer solution (PBS), acetonitrile (ACN) and ethanol were purchased from Merck (Darmstadt, Germany). The Tac powder was purchased from Zahravi pharmaceutical company (Tabriz, Iran). The drug-free plasma was provided by blood transfusion research center (Tabriz, Iran).

### 2.2 Apparatus

UV–Vis absorption spectra were obtained by Shimadzu UV-2450 UV–visible spectrophotometer (Tokyo, Japan). Zeta potential, dynamic light scattering (DLS, Malvern particle size analyzer, Malvern, UK) and field emission scanning electron microscopy (FESEM) images (FEG-SEM MIRA3 TESCAN, Brno, Czech Republic) were employed to characterize the size and morphology of the prepared AgNPs, respectively. Also, Fourier transforms infrared (FTIR) spectrum was applied by a Shimadzu model FTIR prestige 21 spectrophotometers (Tokyo, Japan).

### 2.3 Preparation of plasma samples

The drug-free plasma, which was already frozen in polypropylene microtubes, was thawed at room temperature before daily use. The stock solution of Tac (1000 μg/mL) was prepared in ethanol and used for preparation of Tac-spiked plasma samples (100, 10, and 1 μg/mL). In order to precipitate the plasma proteins, both the drug-spiked and blank plasma samples were mixed by ACN with an equal volume of plasma. The samples were vortexed for 1 min to ensure the denaturation procedure and then centrifuged at 6500 rpm for 15 min. The clear supernatant was discarded and utilized for the colorimetric detection process.

Real plasma samples were obtained from patients with Tac consumption regimens who had signed consent forms with the ethical committee approval code (IR.TBZMED.REC.1400.970). These samples were frozen at −20 °C and melted at room temperature.

### 2.4 General process for tac determination in plasma

A one-step colorimetric method was set for the detection of Tac in plasma samples. To do, Tac-spiked plasma was prepared in different concentrations (0,1,10, and 100 μg/mL) and then the protein precipitation step was performed. Then, about 10 μL of NE (5 mM) and 10 μL of in different concentrations were mixed in a 2 mL microtube and retained for 1 min. In another microtube, a mixture of 50 μL AgNO_3_ (10 mM) mixed by 10 μL NaOH (0.1 M) which causes the color of the solution turn to very pale yellow. Then 150 μL phosphate buffer (50 mM, pH 10) and 800 μL deionized water were added. This solution was mixed by the NE-Tac mixture sequentially which leads to color alteration. In presence of Tac the solution color was altered to brown while it is bright yellow in absence of Tac. The obtained solution was vortexed for 30 s and then it was used for UV–Vis spectra records. The same approach was used for blank samples to compare with Tac-spiked results.

### 2.5 Method optimization process

The quantification process may affect by reaction condition such as pH, vortex time, and amount of AgNO_3_, NE and NaOH. The optimization of these parameters is necessary factor to achieve a sensitive and specific analysis method. As the most important factor, pH was adjusted with phosphate buffer which is prepared by dissolving of disodium hydrogen phosphate in deionized water (50 mM). Then, it was adjusted in pH range of 3–11 by NaoH (0.1 M) and HCl (0.1 M). Also, the adequate volume of phosphate buffer was checked due to addition of 150, 250, 500, and 750 μL with final volume of 1000 μL. The amount of AgNO_3_ (10 mM) was investigated in five volumes of 20, 40, 50, 75 and 100 μL. The effect of the amounts of NE (5 mM) and NaOH (0.1 M) were tested in five volumes including 10, 20, 30, 40, and 50 μL.

## 3 Results and discussions

### 3.1 Mechanism of colorimetric detection

**Scheme 1:**
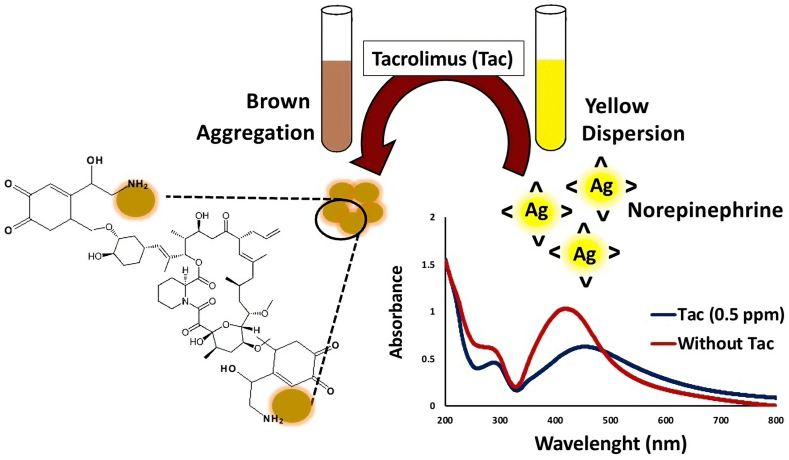
Schematic illustration of the colorimetric detection of Tac via NE-functionalized AgNPs.

It has been confirmed that catecholamines such as dopamine and NE molecules form chemically adherent films on material surfaces through oxidative polymerization under alkaline conditions [41]. Two ortho-located hydroxyl groups of NE transfer two electrons in an oxidative reaction to form a quinone structure on the surface of AgNPs, while the alkylamine groups stabilize the AgNPs [23]. Scheme 1 demonstrates the color and the UV–Vis absorption spectrum alternation in the presence and absence of Tac. Upon addition of Tac, the color of the AgNPs suspension is obviously changed from yellow to reddish brown, proposing aggregation of AgNPs. A main sharp absorption peak of NE-functionalized AgNPs was observed at 410 nm in the absence of Tac, while it turns to a broad peak at 460 nm after the addition of Tac. Due to the Tac-NE binding reaction, AgNPs are aggregated, resulting in a color change. It is noteworthy to mention that no reaction was observed between Tac and AgNPs in the absence of NE and the mixture remained colorless.

### 3.2 Characterization of NE-functionalized AgNPs

**Fig. 1:**
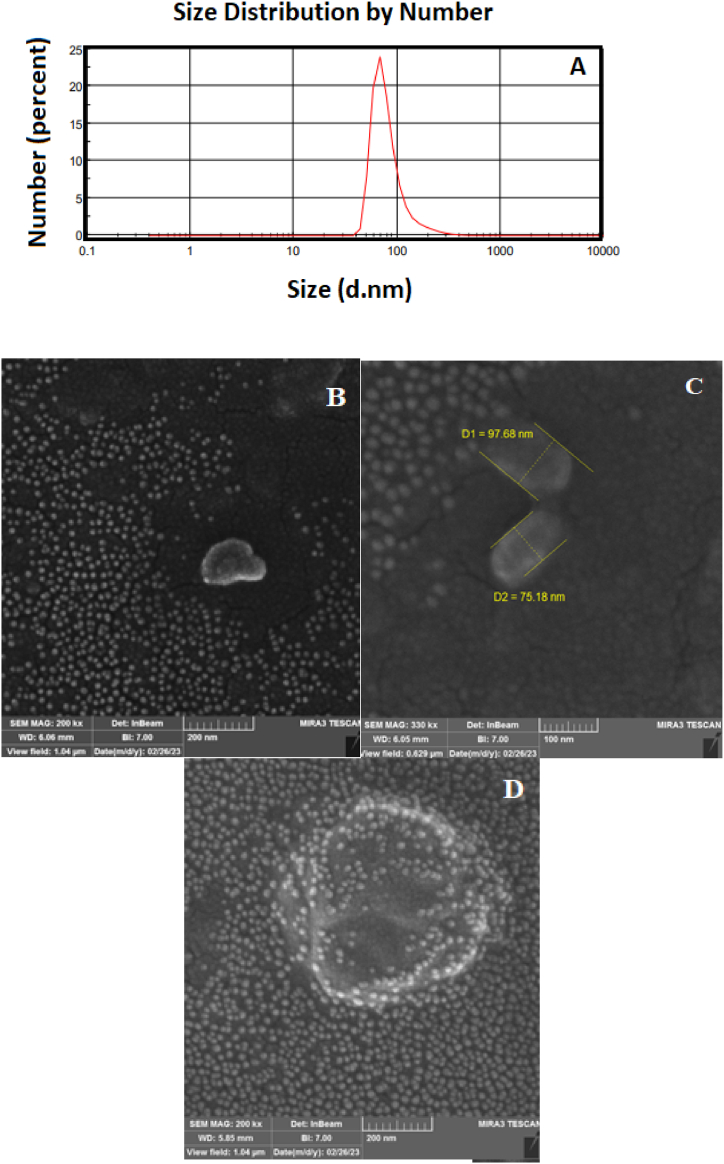
(A) The particle size distribution of the NE-functionalized AgNPs via DLS and (B–D) FESEM images.

Fig. 1A illustrates the size distribution of NE-functionalized AgNPs. The obtained results of DLS indicate the average size of NE-functionalized AgNPs is around 80 nm. Additionally, the SEM images show a large number of tiny spherical particles with an approximate diameter of 20 nm. This can be related to AgNPs (Fig. 1B). Besides, other particles were demonstrated in the SEM images with an average diameter of 80 nm which confirmed the DLS results as the size of NE-functionalized AgNPs. In addition, Fig. 1D demonstrates the aggregation of the AgNPs after the addition of Tac. The elemental composition of NE-functionalized AgNPs measured by EDX analysis represented the C, N, O, and Ag elemental percentages of 41.73%, 31.17%, 15.68%, and 11.42%, respectively (Fig. S1). The surface charge of the nanoparticles was determined by the zeta potential technique with a value of −16.8 mV (Fig. S2), which is close to the catecholamine capped nanoparticles [23].

**Fig. 2:**
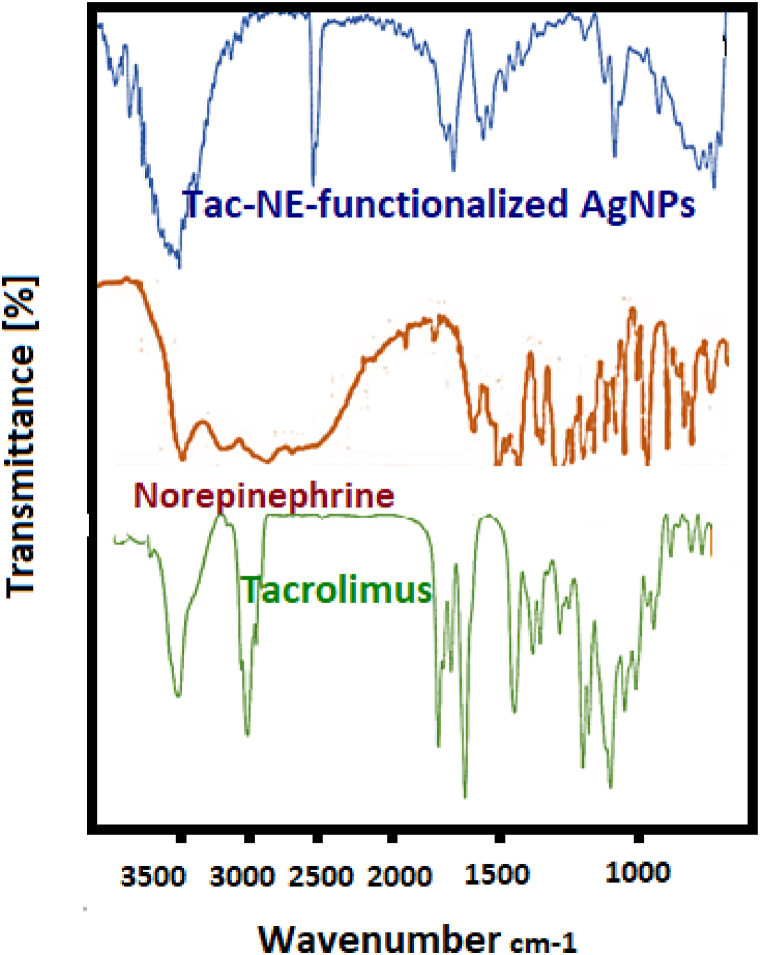
Comparison of FTIR spectra of the NE and Tac with NE-functionalized AgNPs.

The standard FTIR spectrum of NE demonstrates an overlapped band around 3200-3400 cm^-1^ which is assigned to the stretch vibration of NH and OH [42]. These peaks are changed to the sharp peak at 3500 cm^-1^ in the NE-functionalized AgNPs spectrum. This can be related to oxidative reaction of two ortho-located hydroxyl groups of NE and form a quinone structure on the surface of AgNPs [23]. Also, a peak at around 1650 cm^-1^ indicates the carbonyl group (C=O) of the quinone compounds (Fig. 2A). Additionally, the FTIR spectrum of Tac shows a sharp peak at approximately 3000 cm^-1^, related to the alkenyl C–H stretch, which is eliminated at NE-functionalized AgNPs in the presence of Tac. This shows donor-acceptor binding between the alkene groups of the Tac and quinone structures of NE (Fig. 2B).

**Fig. 3:**
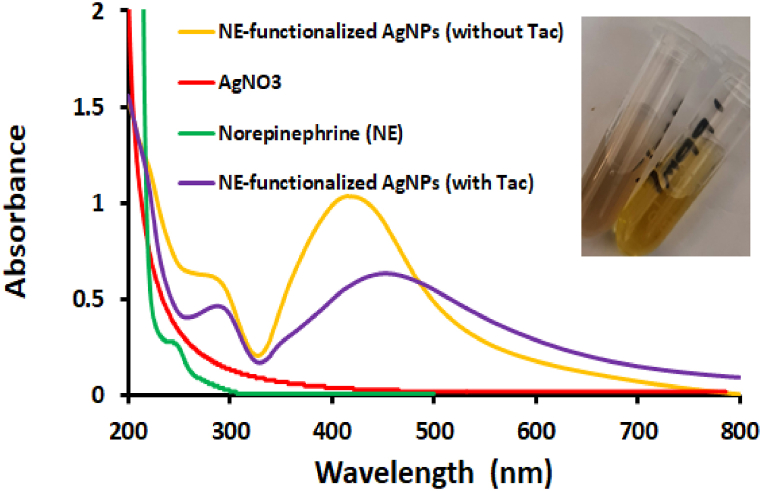
UV–Vis spectra of the AgNO_3_, NE and NE-functionalized AgNPs in presence and absence of Tac.

Fig. 3 shows the absorbance of the NE-functionalized AgNPs in the presence and absence of Tac with the NE and AgNO_3_ spectra. The absorption peak of NE-functionalized AgNPs at 410 nm is changed to 460 nm after Tac addition. Furthermore, NE has a broad absorbance peak around 250 nm, which is evident in the NE-functionalized AgNPs spectrum. The absorbance spectrum of AgNO_3_ has no significant peak in the UV/Vis range [43].

### 3.3 Effect of pH on the absorbance of the NE-functionalized AgNPs

The influence of pH on the absorbance and wavelength of the NE-functionalized AgNPs was investigated. Fig. S3 demonstrates the effect of different pHs which are adjusted by adding 150 μL phosphate buffer in the range of 3.0–10.0. The absorbance increased from pH 5 to 7 and reached its maximum value at pH 8, then decreased up to 10. Some evidence has been reported that metallic NPs are agglomerated at acidic pHs (less than 5) upon neutralization of the surface charges, leading to a change in the SPR of the metallic NPs [44]. In addition, it is observed that the effect of pH on maximum wavelength was different at the tested pH range, in which a blue shift is observable from pH 3 to 10.

### 3.4 Effect of various factors on the tac colorimetric determination

The quantification process of Tac by NE-functionalized AgNPs may be affected by reaction conditions such as pH, vortex time, and the amount of AgNO_3_, NE and NaOH. So, these parameters should be optimized to achieve a sensitive and specific analysis method. To check these phenomena, their effect was investigated by the difference in absorbance of NE-functionalized AgNPs (ΔA = A_1_-A_2_) in the presence (A_2_) and absence (A_1_) of Tac (100 ng/mL).

**Fig. 4:**
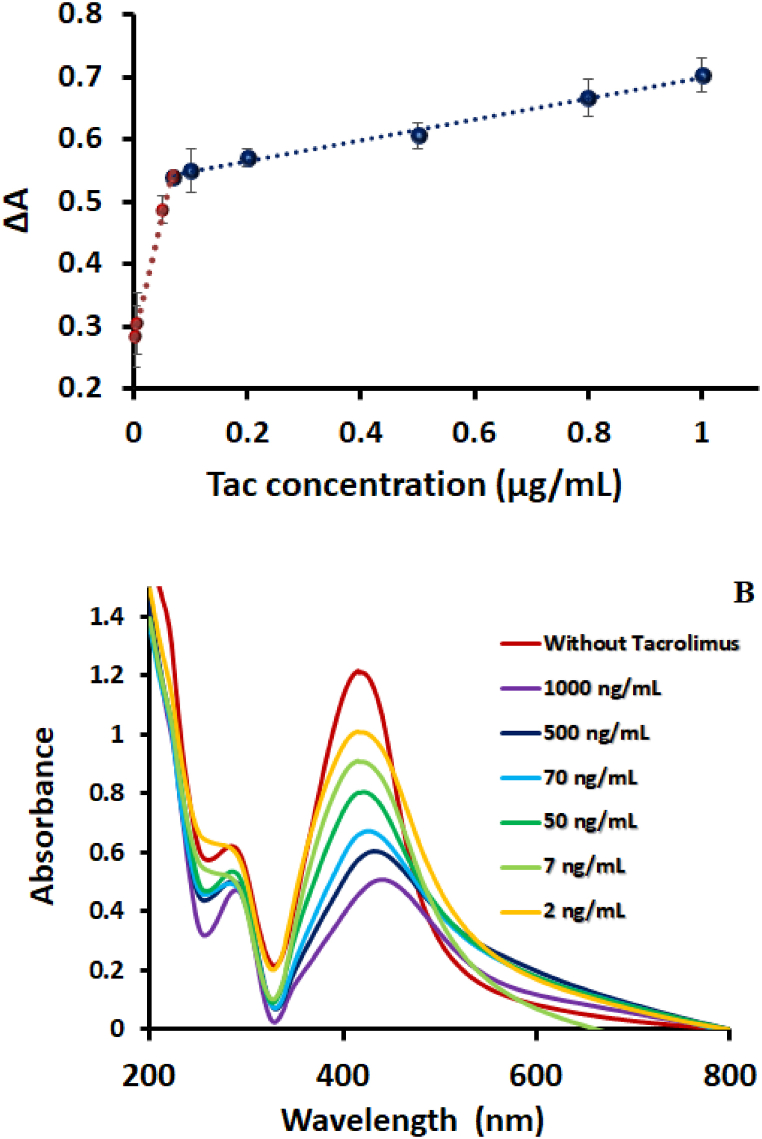
(A) Calibration curve of the Tac determination via NE-functionalized AgNPs and (B) the corresponded absorbance spectra.

The pH value has an important influence on the absorbance intensity and peak maxima, which were studied in the range of 3–11, with a maximum amount of ΔA around pH 10. In acidic pHs (less than 4), the absorbance intensity and peak of the NE-functionalized AgNPs are changed due to the changes in the surface charge of the nanoparticles. The increasing trend was observed gradually in peak intensity from pH 6 to 10. In addition, the volume of PBS was optimized (150 μL) which is important due to its effect on the regulation of the solution ionic strength (Fig. 4S B).

The amount of AgNO_3_ (10 mM) was investigated in five volumes (20, 40, 50, 75, and 100 μL). The amount of ΔA was at its maximum value of 50 μL (Fig. 2S C). The effects of the amounts of NE (5 mM) and NaOH were also tested with an optimized volume around 10 μL where the A_2_ was decreased to its lowest value (Fig. 4S D-E). The higher NE concentrations lead to the formation of less monodispersed AgNPs and a low peak intensity of NPs. The adequate duration of the vortex was observed within 1 min for visual detection of Tac (Fig. 4S F).

### 3.5 Calibration curve and validation

According to the general recommendations of the Food and Drug Administration (FDA), measurements for each typical quantitative analytical evaluation of drugs should be validated by four fundamental parameters include determination of (1) calibration curve, (2) accuracy, precision, and recovery, (3) selectivity, and (4) the stability of analyte in spiked samples. All of these parameters were determined for the proposed method using the calibration curve in plasma samples.

#### 3.5.1 Calibration curve

The calibration curve was constructed via the recording of UV–visible spectra of NE-functionalized AgNPs in eight concentrations of the Tac-spiked plasma samples (2, 7, 50, 70, 200, 500, 800, and 1000 ng/mL) *vis* ΔA. Fig. 4A demonstrates the obtained calibration plot with two linear ranges from 2 ng/mL to 70 ng/mL with an equation of y = 3.8833[Tac] + 0.2782 (p-value <0.001, R^2^ 0.993) and 70 ng/mL to 1000 ng/mL with an equation of y = 0.1695[Tac] + 0.5305 (p-value <0.001, R^2^ 0.993). Fig. 4B describes the gradual decreasing of NE-functionalized AgNPs absorbance intensity by increasing Tac concentration. Meanwhile, a red shift appeared in the maximum wavelength from 410 to around 460 nm, which is observed through a color change from yellow to brown. Limit of detection (LOD) was calculated using 3σ/m and y = 3.8833[Tac] + 0.2782 equation, (where σ is the standard deviation of the blank and m is the slope of the first calibration plot) and it was found as 0.1 ng/mL.

According to the guidelines, the recommended therapeutic blood trough for Tac after kidney transplants should range between 5 and 20 ng/mL [45] and higher levels of 20 ng/mL are directly related to side effects such as nephrotoxicity [46]. Thus, the proposed method not only adequately covers the entire therapeutic range of Tac, but also provides an appropriate platform for the evaluation of toxicities and side effects. A comparison between the performance of the developed method and some of the recent analytical approaches for Tac determination is made in Table 1. The proposed platform quantifies Tac in a suitable linear range, which is comparable with chromatography and MS-based determinations [9,11,15,47]. Although chromatographic methods detect lower concentration of Tac in comparison to the developed method, these methods require extensive training, a time-consuming process, and high-cost sophisticated equipment, making them an inappropriate platform for rapid and real-time routine detection. Also, the colorimetric detection process is capable to observed by naked eyes, which can be considered in smartphone design applications. It is noteworthy to mention that the whole blood samples have a complex matrix, which complicates the detection process more than other biofluids like plasma and pharmaceutical forms [22,48,49]. The obtained analytical figures-of-merit of the developed method indicate that the current method has a potential for rapid and sensitive detection in which the process is as simple as being detected by the naked eye, proposing a promising probe for on-site detection applications. There are only a limited number of spectrophotometric methods available for the quantification of tacrolimus. However, our method is more sensitive and versatile than the previously reported methods. We have also validated the capability of our method in real media and successfully detected tacrolimus in patient samples.

**Table 1:** The analytical figures-of-merit of the studied approaches for the Tac determination.

Technique	Matrix	LOD (ng/mL)	Dynamic range (ng/mL)	Ref.
Coupling of solid-phase microextraction to mass spectrometry	Whole blood	0.3	1–50	[47]
UHPLC	Pharmaceutical Formulation	–	1.0 × 10^5^–3.0 × 10^5^	[15]
LC/MS	Plasma	0.01	0.01–2	[11]
LC-MS/MS	Whole blood	–	1–40	[12]
LC-MS/MS	Whole blood	–	0.5–60	[13]
UPLC-MS/MS	Whole blood	–	50–5000	[16]
HPLC-MS/MS	Whole blood	–	2.25–42.9	[50]
Millifluidic microwave	Whole blood	0.00012	10–500	[51]
Microwave	Blood	0.032	–	[52]
Immunoassay	Blood	–	0.001–1000	[53]
Immunochromatography	Whole blood	0.16	0.48–7.57	[54]
Surface-enhanced raman spectroscopy	Blood	0.33	0.5–20	[55]
Electrochemical immunosensor	Serum	0.17	1–30	[9]
Fluorescent aptasensor	Serum	2.01	6.03–804	[22]
Spectrophotometric	pharmaceutical formulations	–	5.0 × 10^3^-10.0 × 10^3^	[49]
Spectrophotometric	pharmaceutical formulations	–	2.0 × 10^5^-1.8 × 10^6^	[48]
Spectrophotometry	Plasma	0.1	2–1000	This work

#### 3.5.2 Accuracy, precision, and recovery

The precision and accuracy of the analytical method are defined by repeatability (relative standard deviation (RSD%)) and recovery percentage, respectively. The FDA recommends evaluating these values through intra- and inter-day experiments. Three concentrations of Tac-spiked samples, including 3 ng/mL (LQC), 30 ng/mL (MQC), and 90 ng/mL (HQC) were prepared and triplicated. The intra- and inter-day precision values were calculated in the range of 2.32–3.2% and 0.97–4.4%, respectively (Table 2). Also, the recoveries were in the range of 88.78%–105.33% with RSDs less than 5%.

**Table 2:** Accuracy and precision of the developed probe for Tac determination.

Nominal concentrations (ng/mL)	Obtained concentrations (ng/mL)	Intraday precision (RSD%)	Interday precision (RSD%)	Interday accuracy (recovery%)
3.0	2.6	3.2	4.4	88.8
30.0	31.6	5.1	5.5	105.3
90.0	85.6	2.32	0.9	95.1

According to FDA guidelines [56], the evaluated precision and accuracy at each concentration (LQC, MQC, and HQC) should not exceed 15%. So, the obtained results indicated that the developed NE-functionalized AgNPs method had ideal reliability and repeatability for Tac detection in plasma samples.

#### 3.5.3 Selectivity

As a necessity for analytical method validation, the probe selectivity should be investigated to differentiate and determine the intended analyte in the presence of other compounds in the biological samples. The potential interfering substances in a biological matrix may have interacted with probe molecules and altered the results of the analysis.

The selectivity of the NE-functionalized AgNPs probe was studied through the usual co-administrated drugs (mycophenolate mofetil (MMF), azathioprine (AZA), prednisolone (PRDL), and everolimus (Evr), endogenous plasma components (human serum albumin (HSAS), tyrosine (Try), glycine (Gly), cysteine (Cys), glucose (Glu)) and ions (like Zn^2+^, Mg^2+^, Na^+^, Co^2+^, Fe^2+^, K^+^, Cl^−^, Ca^2+^, Al^3+^, and NO_3_^-^). All the interfering agents influence was studied with regard to Table 4 and the Tac concentration was 10 ng/mL.

**Fig. 5:**
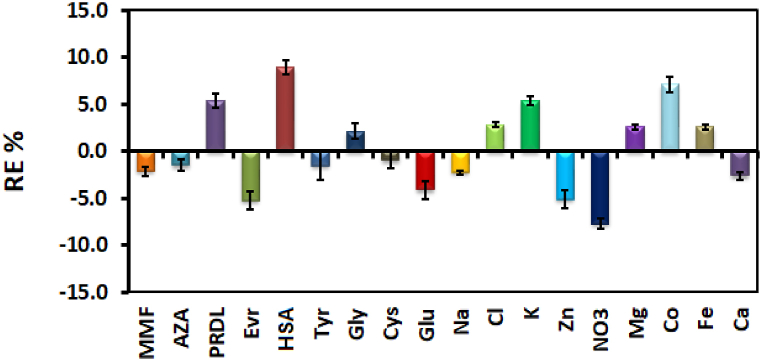
Effect of the common interfering agents on the Tac detection.

As Fig. 5 demonstrates, the RE of all the interfering substances is not as significant as the effect of the detection process (less than 10%), which confirms that the developed method successfully determined Tac concentration in real human samples.

#### 3.5.4 Stability

The stability of the proposed probe was checked by the freeze-thaw method. Three aliquots of two low and high concentrations of Tac-spiked samples (7 and 50 ng/mL) were frozen for 24 h, then thawed at room temperature through three cycles, and then applied to the spectrophotometer. Table 3 provides the obtained results of the stability test, which show the performance of the detection process was reduced by less than 20% after three freeze-thaw cycles.

**Table 3:** Stability test of the proposed method.

Concentration (ng/mL)	Mean found (ng/mL)	Accuracy (RE %)
7.0	5.8	−17.7
50.0	43.6	−12.9

**Table 4:** Effect of the common interfering agents on the Tac detection.

Interfering agents	Conc.(ng/mL)	Recovery%
MMF	20	97.9
AZA	10	98.5
PRDL	20	105.4
Evr	4	94.76
Tyr	80	98.7
Gly	90	98.4
Cys	97	102.2
Glu	850	99.0
Na	100	95.9
Cl	100	97.7
K	100	102.8
Zn	100	105.3
NO3	100	94.8
Mg	100	92.2
Co	100	102.5
Fe	100	107.1
Ca	100	102.5
Al	100	97.4

### 3.6 Application to quantify tac in real plasma samples

Four plasma samples were obtained from patients with a Tac consumption regime who had signed consent forms with the ethical committee approval code (IR.TBZMED.REC.1400.970). Table 5 reports the Tac concentration in the patient’s plasma samples. The number of male and female patients is equal and the administrated dose of Tac for these patients was between 3 and 8 mg per day. In three patients, Tac concentrations ranged in therapeutic index from 4.23 to 19.36 ng/mL.

**Table 5:** Determination of Tac in real plasma samples using the developed method.

Patient (#)	Gender	Measured concentration (ng/mL)
1	Male	13.37
2	Female	4.31
3	Male	26.81
4	Female	19.16

## 4 Conclusion

In conclusion, a one-step and simple colorimetric platform was developed for Tac detection in plasma samples via NE-functionalized silver nanoparticles. It was observed that in the presence of Tac, the solution color was altered, the peak intensity decreased and the maximum wavelength was shifted from 410 to 460 nm. The proposed method offers a facile and fast Tac detection assay that is susceptible for on-site detection applications. Tac concentration was quantified in plasma samples with dual linear ranges from 2 ng/mL to 70 ng/mL and 70 ng/mL to 1 μg/mL with R^2^ > 0.99. This approach holds some benefits, such as high sensitivity and interference-free recognition, and has been successfully applied for Tac determination in four patients under an oral regime as a recommended approach for routine TDM.

## Author contribution statement

Zahra Golsanamlu: Performed the experiments; Wrote the paper.

Jafar Soleymani: Conceived and designed the experiments; Analyzed and interpreted the data; Wrote the paper.

Afshin Gharekhani: Contributed reagents, materials, analysis tools or data.

Abolghasem Jouyban: Analyzed and interpreted the data.

## Data availability statement

Data included in article/supp. material/referenced in article.

## Declaration of competing interest

The authors declare that they have no known competing financial interests or personal relationships that could have appeared to influence the work reported in this paper.

## Abbreviations

Tac: Tacrolimus
NFAT: Nuclear factor of activated T-cells
TDM: Therapeutic drug monitoring
LC-MS/MS: Liquid chromatography tandem mass spectrometry
UPLC-MS/MS: Ultra-high performance liquid chromatography tandem mass spectrometry
ELISA: Enzyme-linked immunosorbent assay
EMIT: Enzyme multiplied immunoassay technique
MEIA: Automated microparticle enzyme immunoassay
ECLIA: Electro-chemiluminescence immunoassay
Au: Gold
Ag: Silver
Cu: Copper
Pt: Platinum
SPR: Surface plasmon resonance
NPs: Nanoparticles
AgNPs: Silver nanoparticles
NE: Norepinephrine
ACN: Acetonitrile
DLS: Dynamic light scattering
FESEM: Field emission scanning electron microscopy
FTIR: Fourier transforms infrared
LOD: Limit of detection
RSD: Relative standard deviation
MMF: Mycophenolate mofetil
AZA: Azathioprine
PRDL: Prednisolone
HSA: Human serum albumin
Try: Tyrosine
Gly: Glycine
Cys: Cysteine
Glu: Glucose
