# Supplementary material for: In-situ preparation of norepinephrine-functionalized silver nanoparticles and application for colorimetric detection of tacrolimus in plasma samples
Source: Heliyon. 2023 Jul 20;9(8):e18404. doi: 10.1016/j.heliyon.2023.e18404 (PMC10412875; doi:10.1016/j.heliyon.2023.e18404)
Supplement: Multimedia component 1 [file mmc1.docx]

**In-situ preparation of norepinephrine-functionalized silver nanoparticles and application for colorimetric detection of tacrolimus in plasma samples**

Zahra Golsanamlu^a,b^, Jafar Soleymani ^a,^^[[1]](#footnote-1)^ , Afshin Gharekhani^a,c^, Abolghasem Jouyban ^a,d^

^a^ Pharmaceutical Analysis Research Center and Faculty of Pharmacy, Tabriz University of Medical Sciences, Tabriz, Iran

^b^ Department of Pharmaceutical Chemistry, Faculty of Pharmacy, Tabriz University of Medical Sciences, Tabriz, Iran

^c^ Department of Clinical Pharmacy (Pharmacotherapy), Faculty of Pharmacy, Sina Hospital, Tabriz University of Medical Sciences, Tabriz, Iran

^d^ Pharmaceutical Sciences Research Center, Shahid Beheshti University of Medical Sciences, Tehran, Iran

**Figures**

**Figure S1**. The elemental composition of NE-functionalized AgNPs declared by EDX analysis


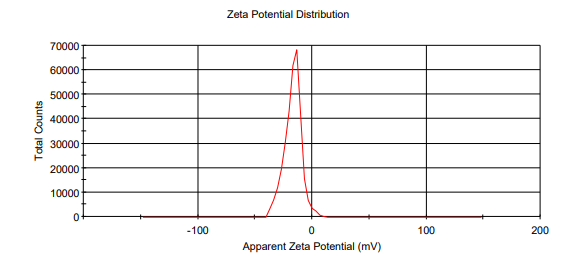


**Figure S2**. Zeta potential distribution of NE-functionalized AgNPs.

**Figure S3**. The effect of different pHs in the range of 3-10 on the UV-Vis spectrum of the NE-functionalized AgNPs.


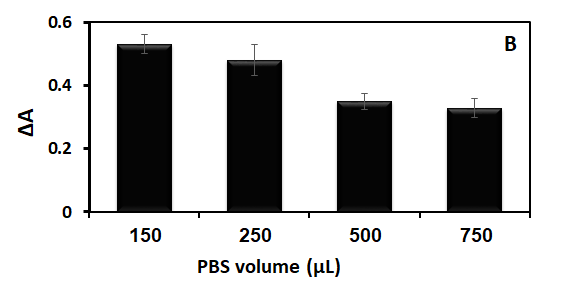

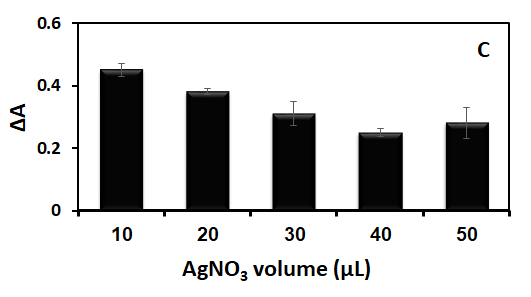


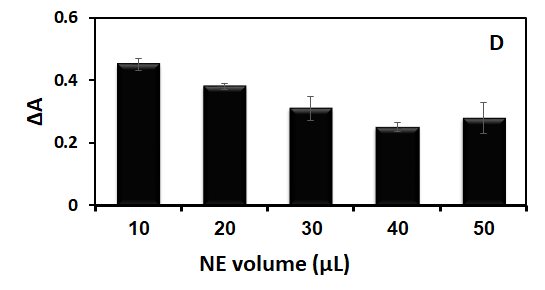

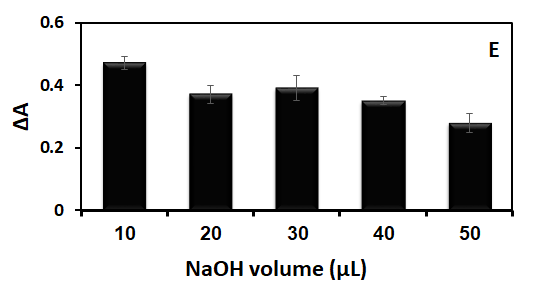

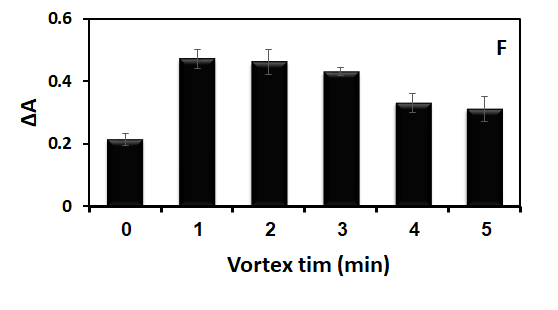


**Figure S4.** The effect of the pH (A), buffer concentration (B), the volume of the AgNO_3_ (C), norepinephrine (D), NaOH (E), and the vortexing time (F) on the Tac detection process by NE-functionalized AgNPs.

1. Corresponding author e-mail addresses: [jsoleymanii@gmail.com](mailto:jsoleymanii@gmail.com) and [soleymanij@tbzmed.ac.ir](mailto:soleymanij@tbzmed.ac.ir), Tel: +9841 33379323. [↑](#footnote-ref-1)
